# Recalcitrance to transformation, a hindrance for genome editing of legumes

**DOI:** 10.3389/fgeed.2023.1247815

**Published:** 2023-09-21

**Authors:** V. M. Nivya, Jasmine M. Shah

**Affiliations:** Department of Plant Science, Central University of Kerala, Kasaragod, Kerala, India

**Keywords:** legume, pulses, transformation recalcitrance, genome editing, genetic engineering, CRISPR/Cas9, plant, virus-mediated genome editing

## Abstract

Plant genome editing, a recently discovered method for targeted mutagenesis, has emerged as a promising tool for crop improvement and gene function research. Many genome-edited plants, such as rice, wheat, and tomato, have emerged over the last decade. As the preliminary steps in the procedure for genome editing involve genetic transformation, amenability to genome editing depends on the efficiency of genetic engineering. Hence, there are numerous reports on the aforementioned crops because they are transformed with relative ease. Legume crops are rich in protein and, thus, are a favored source of plant proteins for the human diet in most countries. However, legume cultivation often succumbs to various biotic/abiotic threats, thereby leading to high yield loss. Furthermore, certain legumes like peanuts possess allergens, and these need to be eliminated as these deprive many people from gaining the benefits of such crops. Further genetic variations are limited in certain legumes. Genome editing has the potential to offer solutions to not only combat biotic/abiotic stress but also generate desirable knock-outs and genetic variants. However, excluding soybean, alfalfa, and *Lotus japonicus*, reports obtained on genome editing of other legume crops are less. This is because, excluding the aforementioned three legume crops, the transformation efficiency of most legumes is found to be very low. Obtaining a higher number of genome-edited events is desirable as it offers the option to genotypically/phenotypically select the best candidate, without the baggage of off-target mutations. Eliminating the barriers to genetic engineering would directly help in increasing genome-editing rates. Thus, this review aims to compare various legumes for their transformation, editing, and regeneration efficiencies and discusses various solutions available for increasing transformation and genome-editing rates in legumes.

## 1 Introduction

Proteins are an integral component of almost every part of our body. The recommended quantity of protein for individuals with minimal to intense physical activity ranges from 1 to 1.6 g per kg body weight per day (Wu, 2016). Legumes contain approximately 13–36 g of proteins per 100 g (Singh et al., 2022; Affrifah et al., 2023). Legumes are also rich in minerals, fibers, and bioactive compounds (Margier et al., 2018). The commonly cultivated grain legumes, also known as pulses, include soybeans, mung beans, field peas, cowpeas, pigeon peas, chickpeas, common beans, and lentils. Legumes are desirable for agriculture as well as they increase the yield of other crops (Zhao et al., 2022) by enhancing soil fertility and nitrogen content. However, legume cultivation suffers an average loss of 31.9%–69.6% due to abiotic (drought) and biotic (insects, diseases, and weeds) reasons (Sharma et al., 2016). Genome editing has recently revolutionized research in crop development as it offers a non-transgenic method of generating targeted mutants with desirable agronomic traits (Bhowmik et al., 2021; Jha et al., 2023; Singh et al., 2023).

## 2 Genome editing

Genome editing is the mutagenesis of desired portions of a gene or genome. Of the various methods used for genome editing, which are based on zinc-finger nuclease (ZFN), transcription activator-like effector nucleases (TALENs), and clustered regularly interspaced short palindromic repeats/CRISPR-associated protein 9 (CRISPR/Cas9) (Amritha and Shah, 2021), CRISPR/Cas9 has proven to be most effective for targeted genome editing in plants (Yin et al., 2017; Mao et al., 2019). CRISPR/Cas9-based genome editing involves site-specific cutting using the Cas9 endonuclease, guided by RNA (Ran et al., 2022). Recently, various versions of Cas (natural and synthetic) and similar nucleases have been reported (reviewed in Aksoy et al., 2022; Liu et al., 2019). Furthermore, various plant promoters for expressing the guided RNAs have previously been reported (reviewed by Kor et al., 2023). This method can be tailored to perform the insertion, deletion, or substitution of nucleotide(s) in the target site (Das et al., 2022; Xie et al., 2022). More details on the process and advances of genome editing have been elaborated in many previous reviews (Aksoy et al., 2022; Ran et al., 2022; Verma et al., 2023b). The mutants of approximately 28 crops including rice, tomato, wheat, and soybean, exhibiting economically important traits such as biotic/abiotic stress resistance and enhanced nutritional value, have been generated in the past few years (reviewed by Verma et al., 2023b; Ukhatovaa et al., 2023). Since transgene integration is not required in the mutants, this method has become the most widely used targeted transgene-free method (Verma et al., 2023b).

Apart from providing abiotic/biotic stress tolerance, genome editing has the potential to eliminate allergy-causing/antinutrient factors from legume crops, such as peanuts (Biswas et al., 2022) and grass peas (Xu et al., 2018; Verma et al., 2023a). Genome editing of legumes can also aid in the functional analysis of genes involved in symbiotic nitrogen fixation (Wang et al., 2017; Wang et al., 2019). Since limited genetic variants are available for cultivated legume crops, genome editing offers an excellent and efficient method for generating favorable mutants. However, there are few reports on legume genome editing due to their transformation recalcitrance.

## 3 Transformation as a prerequisite for genome editing

**FIGURE 1:**
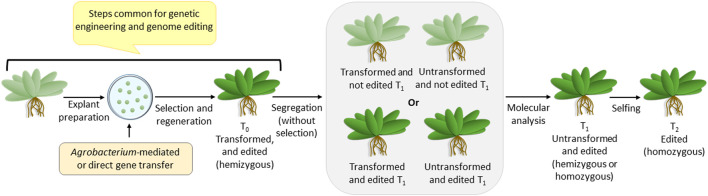
Overview of steps for transformation (also common for genome editing), followed by segregation, leading to a non-transgenic genome-edited plant.

Since the genome-edited plants are non-transgenic, they are generated via steps that are common for making transgenic plants (Figure 1). Like genetic engineering, genome editing reagents are delivered into the plant cell using *Agrobacterium*-mediated or direct gene transfer methods, followed by antibiotic selection and regeneration. Once integrated into the plant genome, the genome editing construct expresses the reagents required for editing and completes the job. Thus, the T0 plants are hemizygous for two loci-—the transformed (harboring the genome editing construct) and the edited loci. Since the segregation of T1 plants would generate the desired homozygous genome-edited candidate, it has to be identified using detailed molecular analysis involving sequencing. The transformed non-edited T1 individuals are eliminated at this stage. The homozygosity of the edited plants is assured by selfing and generating T2 plants. Thus, although genome editing generates a non-transgenic mutant, it can be achieved only if the transformation procedure is followed.

## 4 Transformation recalcitrance of legumes

The main challenge faced during genome editing of legumes is their transformation recalcitrance. Reports on genome editing of plants such as rice and tomato are high (Jaganathan et al., 2018; Ukhatovaa et al., 2023) because they are transformation-amenable. Several crops are susceptible to transformation in comparison to the transformation of recalcitrant crops (Table 1). Table 1 shows that most legumes, excluding alfalfa and *Lotus japonicus*, have lower transformation efficiency. Even soybean, whose genetically modified versions are commercially cultivated in some countries, is known for its recalcitrance to transformation like other legumes (Xu et al., 2022). Only certain cultivars of soybean have generated an appreciable transformation efficiency. Susceptibility to transformation is desirable since it not only reduces the labor of handling more explants but also generates more edited individuals, thereby increasing the probability of obtaining desirable and clean individuals without off-target mutations. For the commercial release of an edited mutant, it is desirable that the best out of many is chosen after thorough genotype/phenotype screening.

**TABLE 1:** Transformation efficiencies of some plants that are susceptible or recalcitrant to transformation.

No.	Type	Plant name	Explant transformed	Transformation efficiency (%)	Reference
1	Susceptible to stable transformation (efficiency >15%)	*Nicotiana tabacum*	Leaf	100	Shah and Veluthambi (2010)
2		*Brassica napus*	Cotyledon	67	Zhang et al. (2005)
3		*Oryza sativa*	Calli	51.77	Raman et al. (2018)
4		*Lycopersicum esculentum*	Cotyledons	41.4	Sharma et al. (2009)
5		*Musa paradisiaca*	Sucker	39.4	Subramanyam et al. (2011)
6		*Hordeum vulgare*	Immature embryo	25	Bartlett et al. (2008)
7		*Zea mays*	Embryo	57.1	Cho et al. (2014)
8		*Brassica juncea*	Leaf	19	Du et al. (2016)
9		Soybean	Seeds	34.6	Karthik et al. (2020)
10		Alfalfa	Leaflets	90	Jiang et al. (2019)
11		*Lotus japonicus*	Seeds	94	Stiller et al. (1997)
12	Recalcitrant to transformation (efficiency < 15%)	*Vigna mungo*	Cotyledonary node and shoot tip	7.6	Muruganantham et al. (2007)
				3.8	Varalaxmi et al. (2013)
			Callus		
13		*Vigna radiata*	Cotyledonary node	4.2	Yadav et al. (2012)
				1.49	Mekala et al. (2016)
			Shoot tip		
14		*Vigna unguiculata*	Cotyledonary node	3.09	Bakshi et al. (2011)
15		*Citrus sinensis*	Epicotyl segments	8.4	de Oliveira et al. (2009)
16		*Citrus paradisi Macf. x Poncirus trifoliate*		11.2	
17		*Malus micromalus*	Leaf segments	6	Zhang et al. (2006)
18		*Cucumis sativus*	Cotyledons	12	Nanasato et al. (2013)
19		Strawberry (*Fragaria* × *ananassa* Duch.)	*In vitro* juvenile leaves	10.8	Zakaria et al. (2014)

### 4.1 Legume transformation

Previously, various explants and regeneration protocols have been attempted in different legume crops (Table 2). As evident in most cases, although the transformation efficiency seemed to be very high when transformed calli were counted based on the expression of reporter genes GUS/GFP/YFP, the number of transgenic plants drastically reduced after the antibiotic-containing media selection. It should also be noted that the true transformation efficiency can be calculated after thorough molecular screening. Although PCR confirms the transgenic nature of plants, the occurrence of clones due to the same transgenic events can be identified after junction fragment analysis by Southern hybridization (Shah and Veluthambi, 2010). Not all previous reports have characterized the junction fragment analysis by Southern hybridization (Table 2). Furthermore, PCR cannot rule out the possibility of transgene amplification due to *Agrobacterium* contamination. Most previous reports do not mention the stable inheritance of the transgene in the subsequent T1 generation. It could be possible that the T0 plants were chimeras and the transgene was lost in the subsequent generation, probably due to an insufficient number of transformed cells per plant.

**TABLE 2:** Details of the transformation and molecular analysis of various legume crops.

No.	Plant name	Explant used	GUS staining	GUS/GFP/YFP expression (%)	Whether regeneration obtained	PCR confirmed in the T0 generation	T0 confirmed by the Southern blot	Details of the Southern blot analysis	Transformation efficiency based on the T0 regeneration (%)	PCR confirmed in the T1 generation	Southern blot confirmed in the T1 generation	Reference
1	*Vigna mungo*	CN	Yes	NM	Yes	Yes	Yes	JF	1	Yes	Yes	Saini et al. (2003)
		SA	Yes	92^b^	Yes	Yes	No	JF	6.5	Yes	Yes	Saini and Jaiwal (2005)
		CN and SA	Yes	76.4^b^	Yes	Yes	Yes	JF	7.6	Yes	Yes	Muruganantham et al. (2007)
		CN	Yes	98^b^	Yes	Yes	Yes	JF	4.31	No	No	Saini and Jaiwal (2007)
		EA	Yes	NM	Yes	Yes	No	NA	2.25	Yes	Yes	Bhomkar et al. (2008)
		Callus	Yes	100^b^	Yes	Yes	No	NA	3.8	Yes	No	Varalaxmi et al. (2013)
		Primary leaf explants	Yes	85^b^	Yes	Yes	Yes	JF	1.3	Yes	No	Sainger et al. (2015)
		Single cotyledon with EA	Yes	46.2^b^	Yes	Yes	Yes	JF	6	No	No	Kapildev et al. (2016)
2	*Vigna radiata*	*Callus, CN*	*Yes*	*95^b^*	*Yes*	*Yes*	*Yes*	*JF*	*0.9*	No	No	Jaiwal et al. (2001)
		Hypocotyl, primary leaves, root, and CN	Yes	80^b^	Yes	No	No	NA	3	No	No	Tazeen and Mirza (2004)
		CN	Yes	88^b^	Yes	Yes	Yes	JF	1.5	Yes	No	Sonia et al. (2007)
		CN	Yes	31.25^b^	Yes	No	No	NA	NM	No	No	Suraninpong et al. (2004)
		CN	Yes	NM	Yes	Yes	Yes	FG	4.2	Yes	No	Yadav et al. (2012)
		SA	Yes	80^b^	Yes	Yes	No	NA	1.49	Yes	No	Mekala et al. (2016)
3	*Vigna unguiculata*	CN	Yes	NM	Yes	Yes	No	NA	0.15	Yes	Yes	Popelka et al. (2006)
		CN	Yes	80^b^	Yes	Yes	Yes	JF	0.76	Yes	No	Chaudhury et al. (2007)
		EA	Yes	25	Yes	Yes	Yes	JF	25	No	No	Raji et al. (2008)
		CN	Yes	100^b^	Yes	Yes	Yes	JF	1.61	No	No	Raveendar and Ignacimuthu (2010)
		CN	Yes	93^b^	Yes	Yes	Yes	JF	3.09	Yes	No	Bakshi et al. (2011)
		Germinated seeds	Yes	90^b^	Yes	Yes	Yes	JF	1.9	Yes	Yes	Kumar et al. (2021b)
		Cotyledonary explant	No	NA	Yes	Yes	Yes	JF	3.47	Yes	No	Kumar et al. (2021a)
4	*Vigna angularis*	Epicotyl	Yes	90.4^b^	Yes	Yes	Yes	JF	2	No	No	Yamada et al. (2001)
5	*Vigna sesquipedalis*	CN	Yes	10	Yes	Yes	Yes	FG	2	No	No	Ignacimuthu (2000)
6	*Cicer arietinum*	EA	Yes	NM	Yes	NM	Yes	JF	0.4	Yes	No	Krishnamurthy et al. (2000)
		EA	Yes	NM	Yes	NM	Yes	JF	3.1	No	Yes	Polowick et al. (2004)
		EA	Yes	74^b^	Yes	Yes	No	JF	26	Yes	Yes	Pathak and Hamzah (2008)
		Epicotyl	Yes	78^b^	Yes	Yes	Yes	JF	24	No	No	Indurker et al. (2010)
7	*Cajanus cajan*	CN and SA	Yes	NM	Yes	Yes	Yes	FG	62	No	No	Geetha et al. (1999)
		Plumule node	No	NA	Yes	Yes	No	JF	15	Yes	Yes	Surekha et al. (2005)
		Axillary meristem	Yes	NM	Yes	Yes	No	NA	65	Yes	Yes	Sharma et al. (2006)
		EA	No	NA	Yes	Yes	Yes	JF	44.6	No	No	Krishna et al. (2011)
		EA-attached cotyledon	Yes	83	Yes	Yes	Yes	JF	83	Yes	No	Karmakar et al. (2019)
8	*Glycine max*	Immature zygotic cotyledon	Yes	100	Yes	No	Yes	FG	0.03	No	No	Yan et al. (2000)
		HSC	Yes	NM	Yes	No	Yes	JF and FG	8.7	No	Yes	Paz et al., 2006
		Cotyledon and hypocotyl	Yes	90	No	Yes (calli)	No	NA	NA	NA	NA	Ismael and Antar (2014)
		CN	Yes	85.7^b^	Yes	Yes	No	NA	6.71	No	No	Jia et al. (2015)
		CN and HSC	Yes	96^b^	Yes	Yes	No	NA	10.01	No	No	Li et al. (2017)
		HSC	Yes	96	Yes	Yes	No	NA	2.5	No	No	Yang et al. (2019)
		HSC with partial EA	No	19.3	Yes	Yes	Yes	FG	18.7	Yes	Yes	Pareddy et al. (2020)
		EA	Yes	66^b^	Yes	Yes	No	NA	22.9	Yes	No	Wang et al. (2022)
9	*Arachis hypogea*	Immature cotyledon	No	NA	Yes	Yes	Yes	FG	48	NM	Yes	Singsit et al. (1997)
		Epicotyl	Yes	42^b^	Yes	Yes	Yes	FG	NM	No	No	Egnin et al. (1998)
		De-embryonated cotyledon	No	NA	Yes	Yes	Yes	JF	17	Yes	No	Tiwari et al. (2008)
		CN	Yes	1.25	Yes	Yes	Yes	FG	2.43	No	No	Hseih et al. (2017)
		HSC	Yes	33.6	Yes	Yes	Yes	JF	33.6	Yes	No	Karthik et al. (2018)
10	*Lens culinaris*	Half embryo	Yes	41.2^b^	No	No	No	NA	NA	NA	NA	Lurquin et al. (1998)
		CN	Yes	99.3^b^	Yes	Yes	Yes	JF	74	Yes	No	Celikkol Akcay et al. (2009)
11	*Pisum sativum*	Half embryo	Yes	54.9^b^	No	No	No	NA	NA	NA	NA	Lurquin et al. (1998)
		EA segments	No	NA	Yes	Yes	No	NA	7.89	No	No	Aftabi et al. (2018)
12	*Medicago truncatula*	Flowers and seedling	No	NA	Yes	No	Yes	FG	76.4	NM	Yes	Trieu et al. (2000)
		Leaflets	Yes	NM	Yes	Yes	No	NA	24	NM	No	Chabaud et al. (2003)
		Root and hairy root	No	NA	Yes	Yes	Yes	JF	41.3	Yes	No	Crane et al. (2006)
13	*Lathyrus sativus* L.	Epicotyl segment	Yes	36.25	Yes	No	Yes	FG	30	NM	NM	Barik et al. (2005)
14	*Lotus japonicus*	Hairy root	Yes	NM	Yes	Yes	No	NA	94	No	No	Stiller et al. (1997)
		Hypocotyl	Yes	NM	Yes	Yes	Yes	FG	1.3	No	No	Kimura et al. (2015)
15	*Lotus corniculatus*	hairy roots	Yes	92	Yes	Yes	Yes	JF	91.67	No	No	Jian et al. (2009)
16	*Phaseolus vulgaris* L.	Leaves and stems	No	NA	Yes	Yes	No	NA	17.4	No	No	Nifantova et al. (2011)
		EA	No	NA	Yes	Yes	No	NA	4.15	Yes	No	Ramírez Rivera et al. (2016)
		EA	No	NA	Yes	Yes	No	NA	2.5	Yes	No	Song et al. (2020)
		EA	Yes	NM	Yes	Yes	No	NA	NM	Yes	No	Sağlam Yılmaz et al. (2022)
17	*Phaseolus acutifolius* L.	EA	Yes	NM	Yes	Yes	Yes	JF	NM	Yes	Yes	Zambre et al. (2005)
18	*Vicia faba* L.	EA	No	NA	Yes	Yes	Yes	JF	2	NM	NM	Hanafy et al. (2005)
		EA	No	NA	Yes	Yes	Yes	JF	1.5	Yes	Yes	Hanafy et al. (2013)
		EA	No	NA	Yes	Yes	No	NA	NM	No	No	Gorji et al. (2014)

^a^ Performed to rule out possible *Agrobacterium* contamination.

^b^ Inclusive of transient expression; JF, junction fragment; FG, full gene; NA, not applicable; NM, not mentioned; CN, cotyledonary node; HSC, half-seed cotyledon; EA, embryonic axis; SA, shoot apex.

## 5 Genome editing of legumes

The availability of deeper information on the whole genome sequences and functional characterization of various genes in many legumes, including soybean, pigeon pea, chickpea, groundnut, common bean, mung bean, and cowpea (Shunmugam et al., 2018; Varshney et al., 2018) has opened vistas for crop improvement via genome editing (Kingsley et al., 2022). Examples of genome-edited legume crops are limited, and these include soybean (Sun et al., 2015; Lu and Tian, 2022), *Lotus japonicus* (Wang et al., 2017), *Medicago truncatula* (Meng et al., 2017; Jaudal et al., 2022), cowpea (Ji et al., 2019; Bridgeland et al., 2023), peanut (Yuan et al., 2019), and chickpea (Badhan et al., 2021; Gupta et al., 2023). Details on the traits of genome-edited legumes are elaborated in previous reviews (Bhowmik et al., 2021; Baloglu et al., 2022; Rasheed et al., 2022). The compilation of the previous reports (Table 4) indicates that although the genome editing efficiency in most cases was appreciably high in the T0 generation, most of these reports do not mention the inheritance of the edited trait in the T1 generation. Furthermore, it is to be noted that the editing efficiencies mentioned in most of these reports were based on the molecular characterization of the callus tissue and not the number of genome-edited T0 individuals. Most of these reports have not mentioned the exact number of edited T0 individuals obtained. This situation is comparable to the genetic transformation of legumes (Table 2), where the efficiency of obtaining GUS/GFP-positive calli was very high but not the transgenic individuals, and importantly, not the transgenic T1 individuals.

## 6 Causes for transformation recalcitrance

**FIGURE 2:**
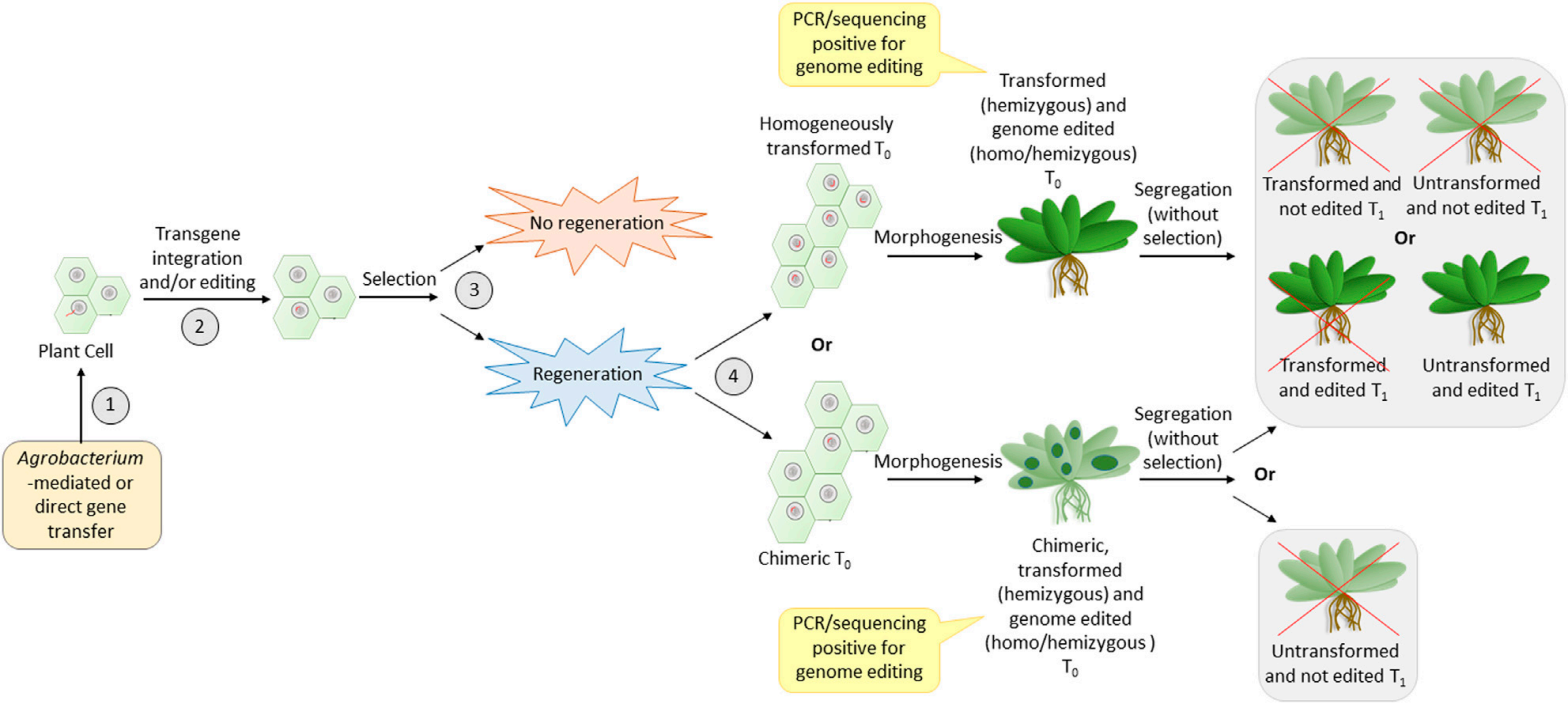
Most crucial steps of transformation that have an impact on the efficiency of genome editing. Numbers 1, 2, 3, and 4 are the steps—entry of DNA, integration accompanied by editing, selection, and regeneration of transformed calli, respectively. The red line entering into the nucleus and present inside the nucleus is the T-DNA. Red crosses indicate undesirable events.

In order to identify the cause for transformation recalcitrance, it is important to recall the major steps in transformation (common for the direct or *Agrobacterium*-mediated method). Three major turning points are crucial for successful transformation (Figure 2). The first one is the effective entry of foreign DNA into the plant cell and its nucleus. The second is transgene integration, during which the cell is transformed. Editing can also take place in this step if the construct for the same is carried out. The third is the regeneration of transformants under selection pressure. The efficiency of the reporter (GUS/GFP/YFP) expression is a reflection of the efficiency of the first two steps. Thus, a high percentage of reporter expression observed in previous data (Table 2) indicates that the first two steps are successfully achieved in legumes. The transformation efficiency based on the T0 regeneration was extremely low in most of the previous cases. This indicates that the problem could be in the third step, which is regeneration under selection pressure. It appears that regeneration from the transformed cells does not occur frequently. Hence, although the high transformation of calli/explant indicates good transformation susceptibility in most cases, the failure of regeneration of transformed cells decreases the overall transformation efficiency. Nevertheless, the *in vitro* regeneration efficiency of most legumes is quite high (Table 3), when not subjected to transformation. This is an indication that the selection pressure during transformation adversely influences the regeneration efficiency in legumes. The reason for this remains unknown. Much standardization can make the recalcitrant legume crops more amenable to transformation (Bekalu et al., 2023) like their transformation-friendly cousins, alfalfa and *Lotus japonicus* (Table 1).

**TABLE 3:** Regeneration efficiencies and explants used in various legume crops.

No.	Plant name	Explant used	Regeneration efficiency (%)	Reference
1	*Vigna mungo*	Leaf petiole	95	Sainger et al. (2015)
		Callus from cotyledon	68.3	Adlinge et al. (2014)
2	*Vigna radiata*	Immature cotyledon	79.3	Tivarekar and Eapen (2001)
		Leaf	85	Devi et al. (2004)
3	*Vigna unguiculata*	Plumule	100	Aasim et al. (2009)
4	*Cajanus cajan*	Seed	95	Sharma et al. (2006)
5	*Arachis hypogea*	Cotyledon	91.5	Tiwari et al. (2008)
6	*Glycine max*	CN	93.5	Radhakrishnan et al. (2009)

Another observation with legumes is that the majority of reports do not show the inheritance of the transgenic (Table 2) or the edited loci (Table 4) to the T1 generation. This reduced heritability of the transgene/edited loci could probably be associated with the fourth step (Figure 2), where the regenerated plants could either be homogenously transformed or chimeras made of transformed and non-transformed tissues. The problem with chimeric plants is the unassured transfer of desired loci to the gametes. This is because the development of gametes with the desired loci depends on the development of floral meristem from transformed somatic cells, which, in turn, is proportional to the number of transformed cells in the regenerated plant. Hence, even if the first three steps are crossed, the fourth step may be a challenge in most legumes. To overcome this problem, it is better to generate plants via somatic embryogenesis and not via direct/indirect regeneration from calli/explant (Chandra and Pental, 2003). This is because somatic embryogenesis generates true-to-type clones (Gaj, 2004; Krishna et al., 2016). This can be achieved by standardization of the tissue culture medium and careful microscopic observation of the regenerating tissue to ensure the selection of somatic embryogenesis (Chandra and Pental, 2003; Pratap et al., 2018).

**TABLE 4:** Reports on genome editing of various legume crops.

Name of the plant	Explant used for editing	Gene delivery method	Mutagenic efficiency in T0 (%)	Single/multiple target	Edited/targeted gene	Mutation confirmed in the T1 generation	Reference
*Glycine max*	Embryonic callus	Particle bombardment	76	Multiple	DD20 and DD43	Yes	Li et al. (2015)
	Cotyledon	*Agrobacterium rhizogenes*	36.7	Single	bar	No	Cai et al. (2015)
			93.3	Multiple	GmFEI2 and GmSHR		
	Cotyledon	*Agrobacterium rhizogenes*	91.7	Single	FAD2-A and Glyma10g42470	No	Duan et al. (2021)
			36.6	Multiple			
	Seedling	*Agrobacterium rhizogenes*	67.65	Multiple	GmIPK1 and GmIPK2	No	Carrijo et al. (2021)
*Vigna unguiculata*	Seedlings	*Agrobacterium rhizogenes*	67	Single	SYMRK	No	Ji et al. (2019)
	Seed	*Agrobacterium tumefaciens*	0.5	Multiple	VuSPO11-1	No	Juranic et al. (2020)
	Embryonic axis	*Agrobacterium tumefaciens*	68.6	Multiple	Vu-SPO11	Yes	Che et al. (2021)
*Arachis hypogea*	Protoplast	PEG transfection	39.1	Multiple	ahFAD2	No	Yuan et al. (2019)
	Seedlings	*Agrobacterium rhizogenes*	44	Multiple			
	Seedlings	*Agrobacterium rhizogenes*	80	Multiple	AhNFR1 and AhNFR5	No	Shu et al. (2020)
	Protoplast	PEG transfection	0.8	Multiple	Ara h 2	No	Biswas et al. (2022)
	Seedlings	*Agrobacterium rhizogenes*	50	Multiple	AhFAD2	No	Neelakandan et al. (2022)
*Cicer arietinum*	Protoplast	PEG transfection	77.3	Multiple	4CL and RVE7	No	Badhan et al., 2021
	Cotyledon	*Agrobacterium tumefaciens*	78	Single	CaPDS	No	Gupta et al. (2023)
*Pisum sativum*	Seedlings	*Agrobacterium rhizogenes*	52.4	Multiple	PsPDS	No	Li et al. (2023)
		*Agrobacterium tumefaciens*	18				

## 7 Strategies to enhance transformation

Many strategies have been previously discussed to improvise crop transformation to aid genome editing (Altpeter et al., 2016). When it comes to legumes, the transformation efficiency is directly proportional to regeneration efficiency. It is known that regeneration depends on multiple factors, such as culture media, hormone concentration, explant, and other supplements. Table 2 shows that many explants have been used in most legumes. Toward this point, soybean sets an excellent example of an explant-dependent enhancement of transformation. Although soybean is considered a crop recalcitrant to transformation (Xu et al., 2022), there are reports where this legume was transformed with an appreciable efficiency. The use of imbibed split seed with an attached partial embryonic axis resulted in 18.7% transformation, in comparison to the average efficiency of 8.7% (Pareddy et al., 2020). Various additives to the cocultivation/selection/regeneration media have been previously used. Another example of success is soybean, where the addition of sodium nitroprusside aided in uplifting the transformation rates up to 34.6% (Karthik et al., 2020). It should also be noted that the cultivar they used was Pusa 9712, which provided an appreciable efficiency of 23% even without adding sodium nitroprusside. On the other hand, Pareddy et al. (2020) used the cultivars, namely, Maverick and 20 proprietary elite, which provided a lesser average transformation efficiency (18.7%). Since the susceptibility to transformation is cultivar-dependent and the choice of cultivar depends on various agronomic conditions, more efforts are needed to standardize cultivar-specific transformation conditions. Although introgression has been used for the inter-cultivar transfer of transgenes, it is laborious and time-consuming. Pareddy et al. (2020) also reported that the *A. tumefaciens* strain EHA105 provided a better result (up to 23.5%) than EHA101 (up to 15.5%). EHA101 was previously reported to transform multiple soybean cultivars, such as Thorne, Williams, Williams 79, and Williams 82 (Paz et al., 2006). In chickpea, the *A. tumefaciens* strain GV3101 resulted in a better transformation efficiency of 17.56%, in comparison with two other strains EHA105 and LBA4404 with 8.54% and 5.43% efficiencies, respectively (Gupta et al., 2023). *Agrobacterium rhizogenes* also resulted in transformation in legumes such as common bean (Li et al., 2022), *Robinia pseudoacacia* L. (Han et al., 1993), and fenugreek (Garagounis et al., 2020).

The overexpression of morphogenetic regulator genes, such as BABY BOOM and WUSCHEL, increased the percentage of *Agrobacterium*-mediated transformation in monocots, like maize, sugarcane, rice, and sorghum (Lowe et al., 2016). This approach enhanced the regeneration efficiency of genome-edited crops as well (Debernardi et al., 2020; Chen et al., 2022). The transformation efficiency is proportional to the regeneration efficiency. The overexpression of maize GOLDEN2, a GARP transcription factor superfamily member that regulates several biological processes and phytohormone signaling pathways in plants, enhanced the regeneration of rice and maize calli by activating chloroplast development (Luo et al., 2023). Similarly, the homologs of GOLDEN2 from legumes (Wang et al., 2013) could help enhance regeneration in legumes as well. Incorporating such growth-promoting factors during transformation for gene editing may resolve the problem of regeneration of transformed cells in legumes.

Legumes serve as a poor host to most strains of *Agrobacterium*; bacteria alternative to *Agrobacterium* can offer a promising solution to enhance transformation events in legumes. Cho et al. (2022) reported the highest transformation efficiency (35%) for soybean using the novel bacteria *Ochrobactrum haywardense* H1, in comparison to two *Agrobacterium* strains AGL1 and LBA4404, with 26% and 12%, respectively. Other non-*Agrobacterium* natural genetic engineers, such as *Ensifer adhaerens* and *Rhizobium etli* (Rathore and Mullins, 2018), should also be explored for legume transformation.

## 8 Transformation-based strategies to facilitate genome editing

Most previously reported strategies on improvising plant genome editing are based on transformation, where transgene is initially integrated and then segregated out (He et al., 2022; Rasheed et al., 2022; Son and Park, 2022). Since most legumes fail to regenerate efficiently under antibiotic selection pressure, the alternative strategy based on reporter expression could be promising. Gao et al. (2016) introduced the novel strategy of expressing the fluorescent reporter mCherry along with the Cas9 construct in *Arabidopsis thaliana* plants. Although their transformation did not involve antibiotic selection, they could visually identify the transformed T1 plants by screening under UV. Similarly, He et al. (2022) proposed the use of the pigment-based RUBY reporter for genome editing as it gives a reddish coloration to the plants. Unlike the popularly known GUS reporter, RUBY does not require additional substrate/chemicals and can be used for live-screening of plants; unlike mCherry/GFP/other fluorescent reporters, its screening does not require UV (He et al., 2020). Thus, during legume transformation, if we use the gene coding for RUBY instead of the usual antibiotic marker gene, we may obtain more transformants due to the absence of antibiotic selection pressure. The non-transformed T1 plants can easily be segregated based on visual selection and subjected to molecular confirmation to identify the edited candidates.

## 9 Strategies for genome editing bypassing transformation

Deconstructed viral vectors have successfully been used for gene function analysis by silencing in legumes (Constantin et al., 2004; Zhang and Ghabrial, 2006) and other plants (Peyret and Lomonossoff, 2015). However, virus-mediated transformation is not a desirable method for stable transformation as it does not generate transformants that can inherit the transgene. Ironically, the lack of transgene integration has appeared to be a desirable feature for genome editing in plants. There are many recent reports on virus-mediated genome editing in *Nicotiana benthamiana* and a few other plants (Varanda et al., 2021; Zhang et al., 2022). There are no such reports on any legumes except for soybeans (Luo et al., 2021). However, although successful editing was demonstrated in this report, the edited plants were not generated. The major drawback observed in most of the previous reports utilizing the virus-mediated method, including the one on soybean, is that they deployed using the *Agrobacterium*-mediated method to generate Cas9-expression. This is because most viruses fail to cargo the Cas9 construct due to its large size for coding approximately 1,368 amino acids (aa). To tackle this situation, two solutions have been proposed in previous reviews (He et al., 2022; Zhang et al., 2022): one is to use nucleases with small coding regions such as CasΦ U (786 aa), Cas12f1 variants (400–600 aa), TnpB (400 aa), and IscBs (approximately 400 aa). The other is to use viruses that can carry longer constructs such as the *Potato virus X*, *Barley yellow striate mosaic virus*, and *Sonchus yellow net virus* (He et al., 2022; Zhang et al., 2022). Since legumes are hosts to a large number of viruses (Chatzivassiliou, 2021; Jha et al., 2023), their deployability, as carriers of genome-editing reagents, needs to be assessed. Previously, the pea early-browning virus of the pea plant was successfully used for heterologous genome editing via the CRISPR/Cas9 system in *Nicotiana* and *Arabidopsis* plants (Ali et al., 2018). Furthermore, the viral vectors previously used for gene silencing in legumes can be modified for carrying the constructs for genome editing. Some more lengths can be reduced by using a bidirectional promoter that would express both Cas9 and sgRNA (Ren et al., 2019).

Complementing the virus-based genome editing, we use mobile sgRNAs that can move to the apical meristem because these are augmented with sequences promoting cell-to-cell mobility (Ellison et al., 2020). Editing in the apical meristem is desirable because it gives rise to the floral meristem, thereby enhancing the chance of heritability of the edited loci. Furthermore, the genome editing efficiency of legumes is increased by enhancing the expression of the editing reagents by codon optimization and the use of efficient promoters, preferably from the same or related species (Baloglu et al., 2022). Many examples of these have been previously reviewed (Gu et al., 2021; Das et al., 2022; Verma et al., 2023a). When it comes to legumes, a higher genome editing efficiency of chickpea was achieved by using chickpea codon-optimized Cas9 and sgRNA driven by the *M. truncatula* U6.1 promoter (Gupta et al., 2023). Although most reports of plant genome editing use CRISPR derived from *Prevotella* and *Francisella1* (Cpf1), Kim and Choi (2021) and Duan et al. (2021) demonstrated an efficient performance of CRISPR tools from other species, such as *Acidaminococcus* sp. and Lachnospiraceae bacterium in the legume crop, soybean.

**FIGURE 3:**
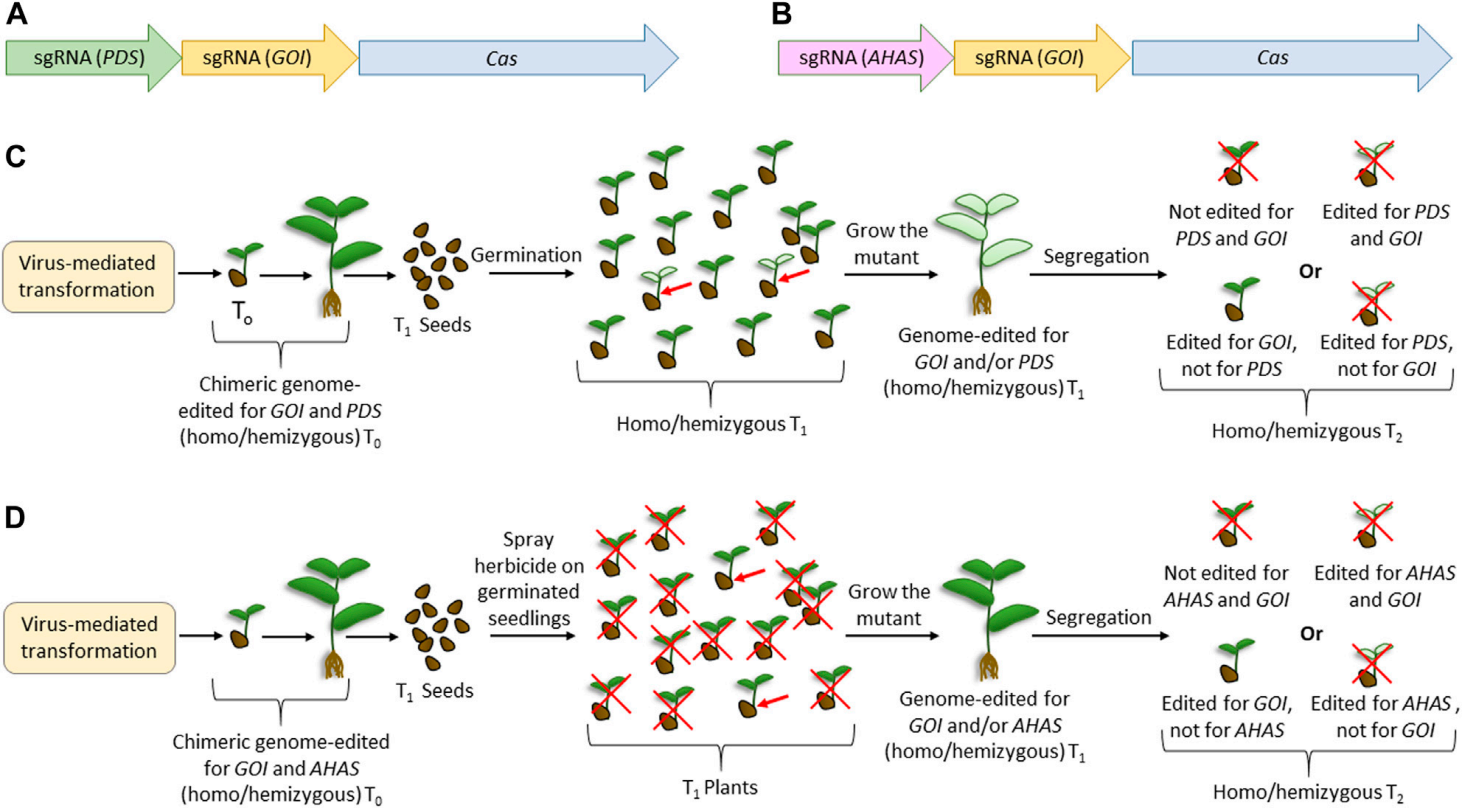
Proposed dual-editing models using the virus-mediated genome-editing method. **(A)** Editing cassette for expressing the sgRNAs of any gene of interest and phytoene desaturase, along with the construct for expressing Cas. **(B)** Editing cassette for expressing the sgRNAs of any gene of interest and acetohydroxy acid synthase, along with the construct for expressing Cas. **(C)** Steps for obtaining plants edited for GOI using the construct expressing sgRNA for PDS. **(D)** Steps for obtaining plants edited for GOI using the construct expressing sgRNA for AHAS. *Cas* is the gene encoding any efficient endonuclease desirable for genome editing. Red crosses indicate undesirable events. Red arrows indicate the expected genome-edited candidate in the experiments.

Combining the previously reported usage of the innate visible marker such as phytoene desaturase (PDS) (Lu and Tian, 2022), herbicide resistance using acetohydroxy acid synthase (AHAS) (Wei et al., 2023), and viruses for genome editing, we proposed two “dual-editing” strategies (Figure 3) that can generate genome-edited legumes, bypassing the transformation step. Here, the viral genome will carry either one of the two constructs (Figures 3A, B). Apart from harboring the expression cassette of a desirable endonuclease (such as Cas9) and the sgRNA for a gene of interest (GOI), the viral genome will carry an additional sgRNA targeting either PDS (Figure 3A) or AHAS (Figure 3B), or genes with similar functions. Thus, the cells harboring the viral replicons can possibly be mutated for both loci. Although the infected T0 plants are chimeric, the flowers emerging from the double-mutant meristematic tissue can set double-mutant T1 seeds. Dual mutation would facilitate the visible selection of plants, thereby reducing the laborious screening through PCR. Thus, in the case where PDS is targeted, T1 will have phenotypes with reduced chlorophyll, which can be selected only by visual screening. Similarly, in the case where AHAS is targeted, the T1 phenotype can be selected after the elimination of unwanted individuals by herbicide application. As various plants have multiple homologs of PDS, the mutant phenotype may vary on a case-to-case basis. For example, two PDS homologs, known as LEAFY (LFY) and KORRIGAN1 (KOR1), are described in pea plants (Constantin et al., 2004). Although the former mutant had bleached leaves and distorted flowers, the inhibition of KOR1 expression significantly reduced shoot/root growth and did not affect flower development (Constantin et al., 2004). In such a situation, it would be desirable to choose the KOR1 homolog of PDS for the dual-editing strategy. Considering the high efficiency of viral-mediated genome editing (Gentzel et al., 2022; Zhang et al., 2022), not only will the possibility of inheritance of the mutant loci increase but there could also be chances of obtaining biallelic mutants for both the loci. The desirable homozygous single mutants for GOI can be segregated by selfing the T1 plants. Although this method can generate the desirable homozygous mutants mostly in the T2 generation, considering the annual life cycle of most legume crops, T2 plants can be obtained in a few months. Importantly, since most legume crops are not susceptible to transgene integration (via biolistic or *Agrobacterium*-mediated), this method could be effective as it does not involve Cas9 integration, as required in most previous reports of virus-mediated editing methods (Zhang et al., 2022). Furthermore, this type of transient expression of Cas9 is desirable because it reduces the off-target mutations due to the reduced time availability in the plant cells. Since the homologs of PDS, AHAS, or genes with similar functions are available in most plants (Hussain et al., 2021), this method of dual editing can have a broad-spectrum application. We believe that similar to this proposed visual screening, it may be possible to utilize other genes with mutants with evident phenotypes, such as the HYPERNODULATION ABERRANT ROOT FORMATION (Har1) (Wopereis et al., 2000), and the temperature-sensitive gene, such as brush mutant (Maekawa-Yoshikawa et al., 2009), of *L. japonicus*.

Previously, Lu et al. (2017) reported a genome editing strategy in rice using CYP81A6, which was analogous to our strategy of using AHAS. CYP81A6 encodes a cytochrome P450 protein, and its silencing renders the plants susceptible to the herbicide bentazon. Thus, Lu et al. (2017) developed a T-DNA-based genome editing vector harboring the Cas9 cassette, sgRNA for a desirable gene, and an RNA silencing construct for CYP81A6. Thus, the transgene-containing T1 plants were visually segregated after the application of bentazon. The two main differences between this strategy and the proposed strategy were that the former involves T-DNA integration and RNA silencing, whereas transient expression and genome editing were involved in the latter. Another similar strategy to the proposed dual-editing is the “dual gRNA” strategy, where one gRNA is aimed for the desirable editing of GOI, and the other is aimed for a large deletion of GOI so that this deletion can facilitate easy screening using PCR (Gao et al., 2016). Here, the homozygous mutants cannot be obtained in T1 since the same loci are differently edited in the same cell. In any case, both analogous methods are not recommended for legumes because they are based on transformation and involve transgene integration, which is not easily accepted by most legumes.

Since Cas9 protein and sgRNA are required for introducing desirable editing into the genome, the integration of the construct expressing these editing reagents is not a prerequisite. Woo et al. (2015) demonstrated the successful genome editing of *A*. *thaliana*, tobacco, lettuce, and rice by delivering the ribonucleoprotein complex comprising Cas9 protein and sgRNA (not the DNA expressing these) into the protoplast using the polyethylene glycol-mediated method. Similarly, Svitashev et al. (2016) and Zhang et al. (2016) edited the genomes of maize and wheat, respectively, by delivering the editing reagents into the protoplast using the biolistic method. Genome editing of the legume crop, soybean, using the ribonucleoprotein transfer via the protoplast culture was previously achieved by Kim and Choi (2021) and Seol et al. (2022). Recently, there have been recommendations for the use of nanoparticles, such as carbon nanotubes, carbon dots, magnetic nanoparticles, and mesoporous silicon nanoparticles, to deliver the ribonucleoprotein complex for editing, by traversing the cell wall (Naik et al., 2022; Verma et al., 2023b). Due to the lack of transgene integration, there is no antibiotic selection in this strategy involving the transport of the Cas9-containing ribonucleoprotein complex. Hence, the major drawback is the laborious PCR-based screening involved since the editing efficiency is quite low and the non-edited individuals outnumbered the edited individuals. We suggest that our “dual-editing” approach can ease the screening procedure. Instead of transporting sgRNAs through the virus (Figure 3), they can directly be introduced into the protoplasts. Since many legumes are amenable to protoplast culture and regeneration (Wiszniewska and Pindel, 2020), it is possible to attain genome editing by directly introducing the editing reagents into the protoplast.

## 10 Conclusion

Extensive standardization of transformation protocols has made soybean, alfalfa, and *L*. *japonicus* amenable to efficient genome editing (Baloglu et al., 2022; Bekalu et al., 2023). Similarly, standardization on other pulse crops should be encouraged. The in-depth research on plant–*Agrobacterium* interaction, regeneration, and development has aided in increasing the transformation in model plants like *Arabidopsis* and *Nicotiana*. It is possible that similar studies in transformation-recalcitrant legumes will aid in rectifying the post-transformation regeneration procedures. Using reporters like RUBY, instead of antibiotic markers, may reduce the adverse effect of selection pressure on regeneration. Methods bypassing transformation, such as the virus-mediated genome editing, could be more promising for legumes. Hence, legume viruses must be analyzed for their capacity to carry the cargo of genome editing reagents. Successful genome editing will aid in incorporating agronomically favorable traits in the legume crops, which serve as an alternative source of protein diet.
